# The role of EGFR mutations in predicting recurrence in early and locally advanced lung adenocarcinoma following definitive therapy

**DOI:** 10.18632/oncotarget.27602

**Published:** 2020-05-26

**Authors:** Carlos Galvez, Saya Jacob, Brian S. Finkelman, Jeffrey Zhao, Kyle Tegtmeyer, Young Kwang Chae, Nisha Mohindra, Ravi Salgia, Borko Jovanovic, Amir Behdad, Victoria Villaflor

**Affiliations:** ^1^Department of Medicine, Northwestern University Feinberg School of Medicine, Chicago, IL, USA; ^2^Division of Hematology-Oncology, Northwestern University Feinberg School of Medicine, Chicago, IL, USA; ^3^Robert H. Lurie Comprehensive Cancer Center of Northwestern University, Chicago, IL, USA; ^4^Department of Pathology, Northwestern University Feinberg School of Medicine, Chicago, IL, USA; ^5^City of Hope Comprehensive Cancer Center, Los Angeles, CA, USA; ^6^Northwestern University, Chicago, IL, USA

**Keywords:** non-small cell lung cancer, lung adenocarcinoma, EGFR, early, recurrence

## Abstract

Introduction: Roughly one third of new non-small cell lung cancer (NSCLC) is diagnosed at early stages. While lobectomy can improve mortality in this group, about 30–55% of patients will experience disease recurrence. Increased investigation into the factors affecting recurrence, particularly tumor molecular genetics such as *EGFR* mutations, is needed.

Materials and Methods: We conducted a single-center retrospective study of 282 patients with early or locally advanced lung adenocarcinoma, with or without *EGFR* mutations, who underwent definitive therapy. We then assessed recurrence, stage at recurrence, time to recurrence and progression-free survival (PFS).

Results: We identified 142 patients with *EGFR*-mutated and 140 *EGFR*-wildtype lung adenocarcinoma. Overall progression between groups was equivalent at ~40% at 5 years; no difference in PFS was observed at any time-point. However, among those who recurred, *EGFR*-mutated lung cancer had increased rates of metastatic recurrence compared to *EGFR*-wildtype disease (97% vs 68%, *p* = 0.007).

Conclusions: *EGFR*-mutated disease may be associated with a higher risk of metastatic recurrence. Molecular testing may be a promising tool for risk stratification and surveillance following definitive management for early stage disease. Future prospective, multi-center cohort studies are needed to confirm these findings and improve our understanding of how *EGFR* mutation contributes to prognosis and clinical outcomes.

## INTRODUCTION

According to the World Health Organization (WHO), lung cancer is the most common cause of cancer and cancer related mortality in both men and women worldwide [1]. Equally important, it is associated with the highest overall mortality responsible for up to ~1,760,00 or 18.4% of all cancer deaths annually [2]. Lung cancer can be divided into small cell lung cancer (SCLC), which accounts for ~15% of cases, and non-small cell lung cancer (NSCLC), which accounts for ~85% of cases [3]. NSCLC can be further subclassified based on histology and presence of driver mutations. Major histologic subtypes of NSCLC include adenocarcinoma (accounting for ~40–50% of all lung cancer cases), squamous cell carcinoma (accounting for ~25–30% of all lung cancer cases), and large cell carcinoma (accounting for ~10–15% of all lung cancer cases) [4, 5].

Lung cancer is typically diagnosed at a late stage due to nonspecific or lack of symptoms (57% of cases with metastatic disease at diagnosis). One-third of newly diagnosed lung cancers present with early stage disease (stage I or II). Furthermore, stage at initial diagnosis is statistically a strong prognostic indicator of survival. Patients with localized disease at diagnosis demonstrate a significantly higher 5-year survival compared to those with metastatic disease at diagnosis (56.3% vs 4.7% respectively) [6]. Complete surgical resection, via lobectomy, with or without adjuvant chemotherapy as appropriate provides the best probability of cure for patients with localized disease. Patients with surgically resectable stage I NSCLC who undergo lobectomy with systematic lymph node dissection have a 5-year survival of ~50–70% [7, 8]. Despite definitive therapy, 30–55% of patients with early NSCLC will eventually experience disease recurrence and die of their disease [9–11]. Across the medical literature, rates of disease recurrence after definitive therapy vary from 30% to 75%, largely depending on stage at initial diagnosis. Median survival in pathologic stage I adenocarcinoma has been estimated between 102–107 months [12]. Furthermore, the majority of recurrences are distant with an average disease-free interval between resection and initial recurrence of 1–2 years [13–15].

With tumor molecular genetics at the forefront of precision medicine, subclassification of NSCLC based on *EGFR* mutation status has been paramount for predicting response to EGFR targeted therapies in unresectable advanced and metastatic disease. It is reported that up to ~20% of patients with NSCLC harbor an *EGFR* driver mutation [16]. Furthermore, according to the PIONEER study, up to 51% of all newly diagnosed untreated Stage IIIB/IV lung adenocarcinoma in Asia harbor an *EGFR* mutation [17]. With the development of targeted therapies, early identification of actionable mutations has revolutionized how we care for patients with unresectable advanced or metastatic disease. The use of anti-EGFR tyrosine kinase inhibitors (TKI) in such populations has been shown to improve overall survival while minimizing treatment toxicity [18].

Despite advances in those with unresectable disease, little is known about the prognostic implications of *EGFR* mutation status in early and locally advanced NSCLC amenable to definitive therapy. While identification of early or locally advanced disease portends more favorable 5-year outcomes, the factors behind the relatively high rates of recurrence are not well understood. Further investigation of molecular tumor markers, particularly *EGFR*, as a predictor of recurrence is required. This single-institution retrospective study aims to better understand the implications of *EGFR* mutation status on localized or locally advanced NSCLC amenable to definitive therapy.

## RESULTS

This study identified 142 patients with *EGFR*-mutated adenocarcinoma who underwent definitive therapy compared to 140 EGFR-wildtype controls. The majority of these EGFR-mutated cases were diagnosed as stage I (107) in contrast to 13 cases of stage II and 22 cases of stage III. Mean age at diagnosis was overall 67 years in both groups and both groups were majority female (104 vs 96 cases respectively). Of the *EGFR*-mutated cases, 35% (*n* = 47) presented with high risk histologic features, defined as visceral pleural invasion, lympho-vascular invasion, poor differentiation, histologic transformation, positive margins, lepidic spread or infarction/necrosis, as noted on final pathology report. This was similar to the number of EGFR-wildtype cases with high risk histologic features (37%, *n* = 42). Clinicopathologic features of patient groups based on *EGFR* mutation status are demonstrated in Table 1.

**Table 1:** Comparison of patient clinicopathologic features based on *EGFR* mutation status

	EGFR mutated	EGFR wildtype	*P*-value
Number of Cases, *N*	142	140	
Stage 1	107	106	
Stage 2	13	13	
Stage 3	22	21	
Age at diagnosis, mean (SD)	67.4 (9.7)	67.2 (9.4)	0.83
Stage 1	67.8 (9.5)	67.3 (9.3)	0.74
Stage 2	70.4 (6.8)	66.1 (11.1)	0.25
Stage 3	63.9 (11.4)	67.0 (9.0)	0.33
Gender—Female, *N* (%)	104 (73%)	96 (69%)	0.46
Stage 1	77 (72%)	72 (68%)	0.62
Stage 2	9 (69%)	11 (85%)	0.64
Stage 3	18 (82%)	13 (62%)	0.26
% with high risk histologic features^*^	47 (35%)	42 (37%)	0.81
Stage 1	29 (28%)	27 (31%)	0.79
Stage 2	8 (62%)	8 (62%)	> 0.99
Stage 3	10 (53%)	7 (54%)	> 0.99
% receiving standard of care^**^	130 (92%)	118 (86%)	0.16
Stage 1	102 (95%)	93 (89%)	0.12
Stage 2	7 (54%)	6 (46%)	> 0.99
Stage 3	21 (95%)	19 (95%)	> 0.99

^*^High risk histologic features = visceral pleural invasion, lympho-vascular invasion, poor differentiation, histologic transformation, positive margins, lepidic spread or infarction/necrosis. ^**^Standard of care defined per NCCN guidelines based on stage of disease. Stage I disease received definitive surgical management and stage II disease received surgery or radiation followed by adjuvant chemotherapy. For stage III standard of care treatment involved multi-disciplinary treatment. Patients with resectable disease received neoadjuvant chemotherapy +/− radiation followed by surgery. For unresectable disease, patients received chemoradiation followed by immunotherapy.

The majority in both groups received standard of care treatment, including cytotoxic platinum-based doublet therapy when appropriate, based on stage (92% vs 86%). Notably, when analyzed by stage, stage II had dramatically fewer cases receiving standard of care treatment (definitive surgery or RT + adjuvant chemotherapy) in both groups (54% and 46% in *EGFR*-mutant and *EGFR*-wildtype groups, respectively). The rationale for this difference was not readily available in this retrospective review, although may be partially explained by the possibility of patients getting adjuvant therapy at outside facilities.

Recurrence after definitive management in early or locally advanced adenocarcinoma occurred in 32 *EGFR*-mutated cases and 29 *EGFR*-wildtype cases. 30 recurrences occurred in patients with stage I disease, 9 recurrences occurred in patients with stage II disease, and 22 recurrences occurred in patients with stage III disease. No significant difference in progression free survival between *EGFR*-mutated and *EGFR*-wildtype cases was found either overall (*p* > 0.99) or at the individual time points of 1, 2, or 5 years (Table 2). We were unable to determine median PFS in the overall cohort, as < 50% individuals in our cohort progressed during the observed follow-up time. However, among those with stage III disease, median PFS was similar at 137 and 167 weeks in the *EGFR*-mutated and *EGFR*-wildtype cases, respectively.

**Table 2:** Comparison of clinical outcomes based on *EGFR* mutation status

	EFGR Positive = 142	EGFR negative = 140	*P*-value
Overall Recurrence (All stage), *N* (%)	32	29	
Stage 1, *N* (%)	18	12	
Stage 2, *N* (%)	2	7	
Stage 3, *N* (%)	12	10	
Metastatic Recurrence among those who recurred, *N* (%)	31 (97%)	21 (68%)	0.007
Stage 1, *N* (%)	17 (94%)	6 (50%)	0.02
Stage 2, *N* (%)	2 (100%)	6 (86%)	> 0.99
Stage 3, *N* (%)	12 (100%)	9 (75%)	0.22
Number of sites of metastasis for those with metastatic recurrence, *N* (%)	1: 21 (68%)	1: 11 (52%)	0.09
	2: 4 (13%)	2: 8 (38%)	
	3+: 6 (19%)	3+: 2 (10%)	
1	21 (68%)	11 (52%)	
2	4 (13%)	8 (38%)	
3+	6 (19%)	2 (10%)	
Progression free survival rates, based on Kaplan-Meier method (95% CI) for All Stages:			> 0.99
1 year (52 weeks)	0.94 (0.89, 0.98)	0.91 (0.86, 0.96)	
2 year (104 weeks)	0.83 (0.76, 0.91)	0.81 (0.74, 0.89)	
5 year (260 weeks)	0.59 (0.47, 0.73)	0.60 (0.50, 0.73)	
Median Progression Free Survival (IQR) in weeks (All stages)^*^	N/A (193, N/A)	N/A (260, N/A)	
Stage 1	N/A (N/A, N/A)	N/A (N/A, N/A)	
Stage 2	N/A (186, N/A)	144 (52, N/A)	
Stage 3	137 (81, N/A)	167 (85, N/A)	

^*^Due to insufficient number of events, point estimates and/or confidence interval bounds were not defined for all cases; these cases are marked as “N/A.”

**Figure 1:**
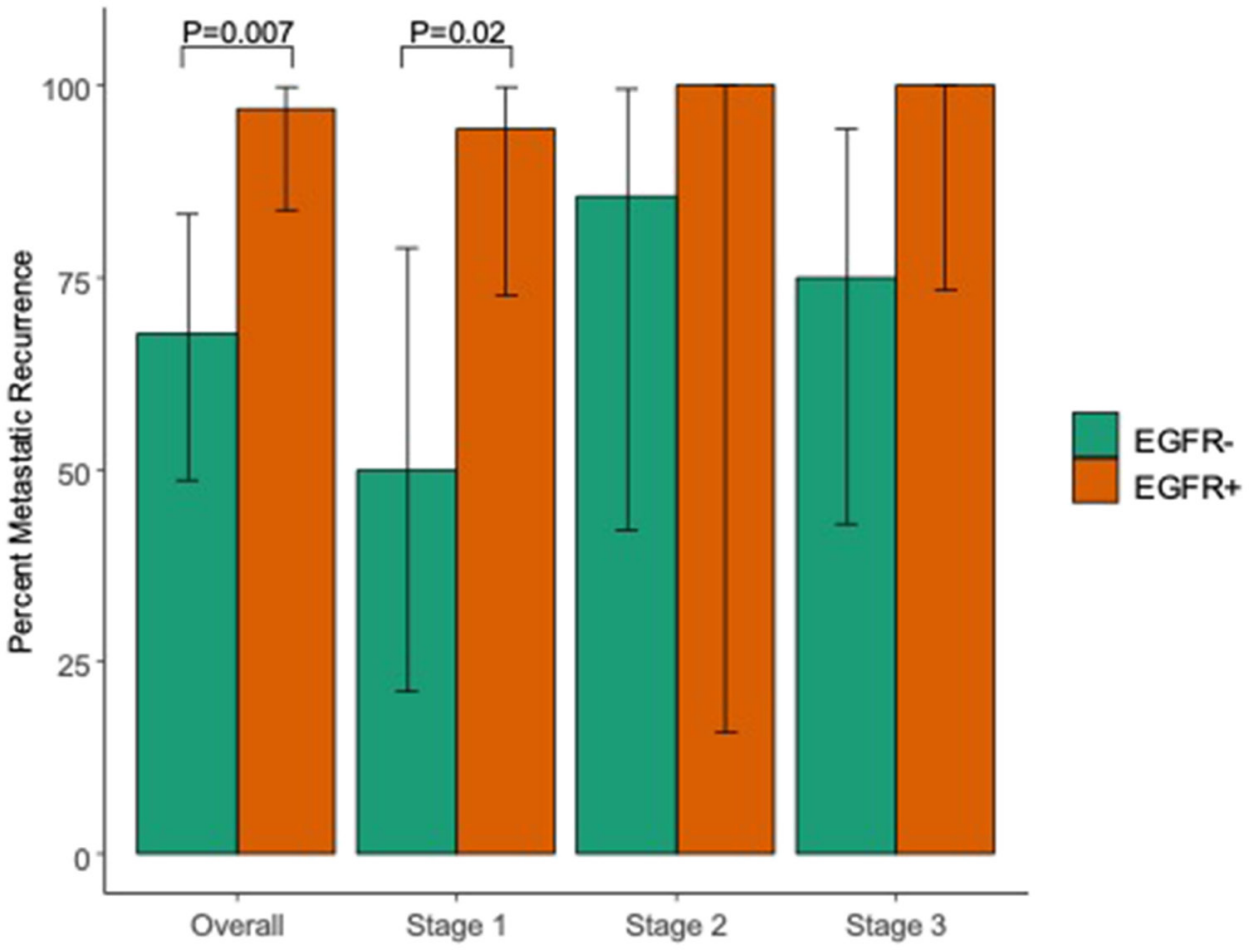
Percent of patients with metastatic disease at recurrence based on initial staging and presence of an *EGFR* driver mutation. Percent metastatic recurrence among those who recurred by *EGFR* status and initial stage.

Our study found that patients with *EGFR*-mutated disease had a marked increase in metastatic recurrence, compared to *EGFR*-wildtype disease (Table 2, Figure 1). Metastatic recurrence was defined as the presence of metastatic disease at time of first recurrence after definitive therapy. More specifically, of those that recurred, 31 (97%) patients with *EGFR*-mutated disease recurred with distant metastasis vs. 21 (68%) with *EGFR* WT (*p* = 0.007). This difference was seen by all patients with early or locally advanced disease who underwent definitive therapy, but when broken down by stage, difference in metastatic recurrence by stage reached statistical significance in stage I disease only (*p* = 0.02). This discrepancy was likely due to fewer cases stage II and III disease resulting in inadequate power to detect differences between groups.

Among cases of metastatic disease recurrence, only a minority of cases in both groups were limited to lung-only recurrence (26% and 14% in *EGFR*-mutated and wildtype disease, respectively, *p* = 0.51). No significant difference was noted between groups with cases of non-pulmonary disease recurrence. The most common sites of distant metastatic disease in the *EGFR*-mutated group included the lung (*n* = 18; 58%), pleura (*n* = 10; 32%), and brain (*n* = 8; 26%). The most common sites of distant metastatic disease in the *EGFR*-wildtype group were lung (*n* = 9; 43%), lymph nodes (*n* = 9; 43%), and brain (*n* = 5; 24%) (Table 3). Although our study was not powered to compare sites of distant metastatic disease, no significant difference was noted in sites of distant metastatic disease between groups.

**Table 3:** Site of metastatic disease recurrence based on *EGFR* mutation status and initial stage

	EGFR Mutated	EGFR Wildtype	*P*-value
Proportion with lung-only metastasis among those with metastasis, *N* (%)	8 (26%)	3 (14%)	0.51
Stage 1	6 (35%)	1 (17%)	0.74
Stage 2	0 (0%)	1 (17%)	> 0.99
Stage 3	2 (17%)	1 (11%)	> 0.99
Metastatic site in those with metastasis, *N* (%)			
Lung	18 (58%)	9 (43%)	0.43
Liver	4 (13%)	1 (5%)	0.62
Bone	6 (19%)	3 (14%)	0.92
Adrenal	1 (3%)	1 (5%)	> 0.99
Brain	8 (26%)	5 (24%)	> 0.99
Pleura	10 (32%)	2 (10%)	0.12
Soft tissue	0 (0%)	1 (5%)	0.84
Lymph node	5 (16%)	9 (43%)	0.07
Thyroid	0 (0%)	1 (5%)	0.84
Gastrointestinal tract	0 (0%)	1 (5%)	0.84
Pericardium	1 (3%)	0 (0%)	> 0.99

Analyses of *EGFR* mutation type were also performed (Table 4). The most common mutations overall were Exon 19 deletions (44%) and L858R (35%). Roughly 70% of both these mutations presented as stage 1; however this may represent the larger number of stage 1 disease within our cohort. Only 2 cases had the T790M mutation and only 5 cases of multiple *EGFR* mutations occurring within one case were noted.

**Table 4:** *EGFR* mutation subtypes

Exon Mutation Subtype	*N* (%)	Stage at Presentation
Exon 19 deletion	63 (44)	Stage 1 = 48 (76%)
		Stage 2 = 7 (11%)
		Stage 3 = 8 (13%)
Exon 20 insertion	7 (5)	Stage 1 = 4 (57%)
		Stage 2 = 0 (0%)
		Stage 3 = 3 (43%)
G719A	2 (1)	Stage 1 = 2 (100%)
		Stage 2 = 0 (0%)
		Stage 3 = 0 (0%)
L858R	49 (35)	Stage 1 = 34 (70%)
		Stage 2 = 5 (10%)
		Stage 3 = 10 (20%)
G719×	6 (4)	Stage 1 = 6 (100%)
		Stage 2 = 0 (0%)
		Stage 3 = 0 (0%)
L861Q	8 (6)	Stage 1 = 7 (88%)
		Stage 2 = 0 (0%)
		Stage 3 = 1 (12%)
T790M	2 (1)	Stage 1 = 2 (100%)
		Stage 2 = 0 (0%)
		Stage 3 = 0 (0%)
Multiple mutations	5 (4)	Stage 1 = 4 (80%)
		Stage 2 = 1 (20%)
		Stage 3 = 0 (0%)

## DISCUSSION

Our study reports a novel finding of increased metastatic recurrence in patients with early and locally advanced *EGFR*-mutated NSCLC undergoing definitive therapy compared to *EGFR*-wildtype disease. The increased rate of metastatic recurrence found is consistent with prior multi-variate analysis showing decreased relapse-free survival in cases of *EGFR*-mutated disease [19, 20]. We did not observe any statistically significant change to survival based on EGFR status (PFS 59% in *EGFR*-mutant vs 60% in *EGFR*-wildtype at 5 years). This supports prior investigation showing no prognostic value of EGFR mutation in early stage lung cancer [21]. Overall our findings suggest that while *EGFR* mutation status does not affect overall recurrence or survival, metastatic vs local recurrence may be influenced by tumor molecular genetics.

The reasons for the high rate of recurrence in early and locally advanced NSCLC after definitive therapy are not completely clear. One theory suggests that most of our standard diagnostic tools for TNM staging, including CT and PET scans, are not sensitive enough to detect distant micro-metastasis present at time of initial diagnoses. This would cause a fundamental under-estimation of tumor burden and extent of disease [11]. Studies have also demonstrated direct evidence of increased tumor cell burden within the pulmonary veins during lobectomy in surgically resectable primary lung cancer, suggesting that surgical manipulation may cause intraoperative tumor cell “spillage” serving as niduses for disease recurrence [22]. Further studies will need to help elucidate the clinical significance of these disseminated tumor cells given the inconclusive evidence of whether these cells actually express a proliferative phenotype versus a “dormant” one [23].

Either of the above mechanisms could help explain the observed higher rates of metastatic recurrence in *EGFR*-mutated disease. For instance, it is possible that the *EGFR*-mutated phenotype results in a unique tumor physiology that results in higher rates of microscopic foci of disease, even at a clinically low stage. These sites of micro-metastatic disease in *EGFR*-mutated tumors likely represents a more systemic disease process in contrast to more localized disease process or field cancerization found in *EGFR*-wildtype disease. An alternative explanation may be that *EGFR* mutated tumors tend to have more tumor cell “spillage” during or prior to surgery, as indicated in prior studies, which in turn results in increased rates of metastatic recurrence [11, 22]. This explanation is further supported by the overwhelming recurrence in lung parenchyma and pleura observed in the *EGFR*-mutated group, as these sites would be most affected by increased tumor cell burden in pulmonary veins. Furthermore, our study was not powered to detect statistically significant differences in sites of metastatic disease between groups. Additional large observational cohort studies are needed to help determine whether the more systemic nature of *EGFR*-mutated tumors affects sites of metastatic disease at time of first recurrence.

This study showed no statistically different progression free survival between *EGFR*-mutated and negative groups. We were unable to calculate median progression free survival time in the overall cohort due to insufficient follow-up time; however, median progression free survival was similar among those with stage III disease. Future study of recurrence rates in early and locally advanced NSCLC involving large prospective and multi-center cohorts is needed to validate these results.

Currently, molecular testing in NSCLC is FDA-approved for metastatic NSCLC, but it is not routinely performed in early and locally advanced disease. Additionally, only radiographic guidelines exist for monitoring disease recurrence following definitive surgical therapy in lung cancer. This may not be sufficient for detecting disease which may be intrinsically more systemic and diffuse in nature [24]. The increased rate of metastatic recurrence in *EGFR*-mutated disease found in this study suggests both distinct tumor physiology as well as a need for earlier use of tumor molecular genetics. These patients may also require more stringent monitoring following curative treatment, as early detection of these recurrences can lead to initiation of treatment. In fact, multiple studies have demonstrated improved post-relapse survival compared to wildtype [19, 25, 26] in *EGFR*-mutated disease, likely due to response to TKI targeted therapies [27].

In summary, this study suggests *EGFR* mutation as an important marker for predicting metastatic disease recurrence and highlights the growing need for precision medicine in early and locally advanced NSCLC. Early identification of these recurrences is paramount given the improved post-relapse survival observed in this population. A better understanding of the factors leading to relapse rates using prospective, multi-center investigations could help guide future surveillance practices, identify those patients at higher risk, and ultimately extend patient survival.

## MATERIALS AND METHODS

### Patients

We retrospectively identified 142 patients between the ages of 18–99 years with pathologically confirmed *EGFR*-mutated adenocarcinoma at the McGaw Medical Center of Northwestern University from 2007 to 2018. These patients were identified based on individual chart review of all NSCLC patients who had positive tumor molecular genetics. Only those patients who had biopsy-proven *EGFR* mutations at time of diagnosis were included. Additionally, patients who were lost to follow-up, presented with metastatic disease, or did not undergo treatment with curative intent at time of initial diagnosis were excluded.

We also randomly identified 140 patients between the ages of 18–99 years with *EGFR* wildtype lung adenocarcinoma as controls. Similar to the *EGFR*-mutated cohort, those included in the cohort underwent tumor molecular genetics at time of diagnosis, had nonmetastatic disease at presentation, and underwent definitive management. Similarly, those who were lost to follow-up, presented with metastatic disease, or did not undergo definitive therapy were excluded from the cohort.

### Molecular assays

All molecular genetic testing was done on samples from formalin-fixed paraffin embedded lung tissue specimens. Macrodissection was used for tumor enrichment when needed to ensure greater than 10% tumor cellularity. DNA extraction and purification was performed on automated nucleic extraction instrument QIAsymphony SP using QIAsymphony DNA Mini Kit (Qiagen, Valencia, CA, USA). Before July 2015, the *EGFR* testing was performed using *EGFR* Mutation real-time PCR Analysis Kit (EntroGen, Tarzana, CA, USA) following the manufacturer’s protocol. After July 2015, *EGFR* mutations were detected using the Ion AmpliSeq Colon and Lung Cancer Research Panel v2. This panel is an amplicon based next generation sequencing assay (NGS), consisting of 22 targeted genes. Sensitivity of detection was set at 5% mutant allele or 10% tumor DNA.

### Clinicopathologic characteristics

We assessed age of diagnosis, gender, NSCLC histology, stage at diagnosis, EGFR status at time of diagnosis, specific EGFR mutation type where applicable, presence of high-risk pathologic features upon surgery and type of definitive treatment. Identified cases were then retrospectively chart-reviewed for presence of pathologically confirmed recurrence, stage of recurrence, time from definitive treatment to recurrence, and overall progression-free survival at 1,2 and 5 years. Stage at diagnosis was calculated using the TNM staging system defined by the American Joint Committee on Cancer 8th edition [28]. Progression was defined as recurrence (local or metastatic) or death by any cause. Progression-free survival was calculated based on Kaplan-Meier methods. Association between categorical variables was performed using chi-square or Wilcoxon sum test where applicable. Patient cases were also evaluated for delivery of standard of care treatment, defined using NCCN guidelines [29]. More specifically, patients with stage I disease received definitive therapy with either surgery or radiotherapy. Stage II disease received surgery or radiation followed by adjuvant chemotherapy. Management of stage III disease involves a multi-disciplinary treatment plan largely depending on surgical resectability. Patients with resectable disease received neoadjuvant chemotherapy +/− radiation followed by surgery +/− adjuvant radiation (if not previously received). For unresectable disease, patients received chemoradiation followed by immunotherapy. All tests were two-sided and *p*-values < 0.05 were considered statistically significant. All statistical analyses were performed using R 3.4.1.
